# Examining sex differences in the completeness of Peruvian CRVS data and adult mortality estimates

**DOI:** 10.1186/s41118-021-00151-5

**Published:** 2022-01-15

**Authors:** Helena Cruz Castanheira, José Henrique Costa Monteiro da Silva

**Affiliations:** Latin American and Caribbean Demographic Centre (CELADE)-Population Division of the United Nations Economic Commission for Latin America and the Caribbean (ECLAC), Santiago, Chile

**Keywords:** Civil registration and vital statistics, Adult mortality, Sex differences, Life expectancy, Peru

## Abstract

The production, compilation, and publication of death registration records is complex and usually involves many institutions. Assessing available data and the evolution of the completeness of the data compiled based on demographic techniques and other available data sources is of great importance for countries and for having timely and disaggregated mortality estimates. In this paper, we assess whether it is reasonable, based on the available data, to assume that there is a sex difference in the completeness of male and female death records in Peru in the last 30 years. In addition, we assess how the gap may have evolved with time by applying two-census death distribution methods on health-related registries and analyzing the information from the Demographic and Health Surveys and civil registries. Our findings suggest that there is no significant sex difference in the completeness of male and female health-related registries and, consequently, the sex gap currently observed in adult mortality estimates might be overestimated.

## Introduction

Civil registration and vital statistics (CRVS) systems are essential to the monitoring of multiple health- and mortality-related Sustainable Development Goals for an effective planning of evidence-based health policies and for the allocation of financial resources (Richards et al., 2018). When CRVS systems are not implemented or are not robust enough for the construction of reliable health indicators, household surveys and population censuses are alternatives (AbouZahr and Boerma, 2005). These information sources, however, lack the required details for some policy-specific situations. For example, several of the Sustainable Development Goals are directly related to causes of deaths, which require reliable information on individual cause of death following the International Statistical Classification of Diseases and Related Health Problems (ICD) (Naghavi et al., 2010). In addition, CRVS systems are able to produce health and mortality indicators that are continuous and at a smaller scale at relatively lower costs, which contributes to monitoring health outcomes in remote and vulnerable areas (The University of Melbourne, 2019).

The importance of CRVS systems has been further highlighted during the COVID-19 pandemic, since countries with reliable systems are able to implement real-time monitoring of total deaths and plan informed health policy approaches to tackle the health crisis (World Health Organization, 2020).

Latin America and the Caribbean (LAC) countries experienced an unprecedented mortality decline in the second half of the twentieth century (Guzmán et al., 2006; United Nations, 2019c). These declines were followed by increases in death registration completeness levels through improvements of CRVS systems in the region (Guzmán et al., 2006; Gonzaga et al., 2018). The quality and coverage of CRVS data is heterogeneous in LAC countries (Guzmán et al., 2006; Palloni and Pinto-Aguirre, 2011; Palloni et al., 2016). For some LAC countries, death counts from administrative records have only recently become available for statistical purposes, disaggregated by age and sex (Mikkelsen et al., 2015). Even when these data were available, they could contain inconsistencies in cause-of-death information, age groups, usual residence, or place of registration (Faijer, 1994; Mikkelsen et al., 2015). Mortality assessments in LAC countries in the past were performed mostly using indirect methods which rely on summary indicators computed from household surveys or population censuses—such as under-5 mortality—and model life tables (Palloni, 1981; Moultrie et al., 2013), although there is a significant difference across countries and CRVS systems (Palloni et al., 2016).

Despite the overall improvements observed in the LAC region since the 1980s, Peru has reported low historical levels of completeness in the number of deaths registered in administrative records (Piscoya-Diaz and Queiroz, 2010) and deficient cause-of-death classification, presenting high proportions of ill-defined deaths (Naghavi et al., 2010). In this sense, the progress of the CRVS system’s performance in the country was classified as slow (Mikkelsen et al., 2015). Nevertheless, the country has made substantial efforts to improve the CRVS systems over the last decade (Vargas-Herrera et al., 2018).

Although the CRVS system in Peru is considered incomplete, its health-related data, which are published periodically by the *Instituto Nacional de Estadística e Informática* (INEI), or National Institute of Statistics and Informatics, is a continuous series by age and sex that spans over three decades. Combining this information with the most recent Population and Housing Census in the country (INEI, 2017) allows us to evaluate the relative completeness of registries for the last decades by sex and regions.^1^ This analysis is useful to shed light on the relative completeness of the health-related registries in the country and regions, and to assess the differential completeness of the registries by sex, illuminating with real data the possible evolution of the sex gap in adult mortality in the country. Despite the recent estimates of completeness of death registration by sex (Piscoya-Diaz and Queiroz, 2010), not much has been discussed about the trends in sex differentials in death registries from the health information system or in the evolution of the sex gap in adult mortality over the last decade in Peru.^2^

The main objective of this paper is to assess the death registration relative completeness (DRRC) by sex over the last three decades in Peru for national and subnational levels. Then, the impact of the estimated DRRC sex differentials on the magnitude of Peru’s observed mortality sex gap is evaluated. The observed trend in the sex gap is compared across multiple data sources and methods of mortality estimation. In this regard, data from the civil registry, Ministry of Health, and national demography and health surveys are used. Finally, a discussion on the sex differential in adult mortality in Peru is presented, considering the existing evidence on the sex gap, highlighting the importance of using existing CRVS data in developing countries to assess mortality differentials.

## Sex differentials in completeness of death registration

Most studies evaluating death registration completeness report its estimates by sex; however, the discussion regarding sex differentials in death registration is still scarce. In general, there are significant cultural and economic gender biases that hinder women’s access to civil registration during delivery, marriage, and death (Silva and Snow, 2019; Orrell, 2020; Cobos et al., 2020). In countries with high levels of gender inequality on birth registration, access to identity cards, and inheritance rights, the deaths of males with assets are usually more likely to be registered (Orrell, 2020).

Recent results of death registration completeness exhibit mixed findings in regard to female disadvantages. Overall, most Latin American countries do not show significant differences in completeness between males and females (Gonzaga et al., 2018). Ecuador, on the other hand, has reported slightly lower levels of death registration completeness and higher numbers of poor-quality death registries for females (Peralta et al., 2019). In India, these numbers are still large, with 11% of female deaths being underreported compared to 5% of male deaths in the 2000–2010 period for its Sample Registration System (Yadav and Ram, 2019).

In Peru, Piscoya-Diaz and Queiroz (2010) and Gonzaga et al. (2018) found no clear pattern of sex differentials in death registration completeness; the male or female mortality advantage varied by region and by age trim selection when applying death distribution methods.

## Sex differentials in life expectancy and mortality

Sex differentials in mortality have been widely studied and are well documented in the literature (United Nations, 1988). In most countries, women currently show higher survival rates than men (United Nations, 2019c). This is caused, among other things, by biological factors (Luy, 2003), different health behaviours of males in relation to females—related to alcohol consumption, smoking, and differences in diet (Beltrán-Sánchez et al., 2015), greater adult male vulnerability to cardiovascular diseases (Preston, 1976), and violence (Canudas-Romo and Aburto, 2019). Among the related causes of death, significant excess male mortality is observed in young adulthood (15–24) (Nathanson, 1984) due to external causes and in late adulthood (50–70) (Preston, 1976; Nathanson, 1984) due to cardiovascular diseases and lung cancer, among other factors.

In the twentieth century, mortality rates decreased faster for females than for males (Beltrán-Sánchez et al., 2015), especially in countries able to significantly reduce maternal mortality and tuberculosis (Waldron, 1985). Although women are more likely to discover illness, because they attend health services more often than men, as women have acquired similar health behaviours (especially related to diet, smoking, and alcohol consumption), the increase in sex differentials began to slow down (Wingard, 1984). The sex differentials narrowed as observed for industrialized countries from 1970 to 1990 (Trovato and Lalu, 1996).

In Latin America, male deaths from external causes (homicides, accidents, and suicides) contributes to the observed differences in life expectancy and years of life lived in adulthood between males and females (Alvarez et al., 2020; Calazans and Queiroz, 2020). Peru also exhibits a relevant impact of external causes on males’ life expectancy, even though to a smaller extent when compared to other LAC countries, such as Colombia, Honduras, and Mexico (Canudas-Romo and Aburto, 2019; Calazans and Queiroz, 2020). Nevertheless, in Peru in 2016, males were more than twice as likely to die from accidents and injuries than females when considering reported causes of deaths (Bernabé-Ortiz and Carrillo-Larco, 2020).

## Data and reference estimates

### Data for adult mortality assessment in Peru

#### Civil registry and health ministry data

Peru has two main sources of administrative registries that provide annual information on deaths: the civil registry (RENIEC, *Registro Nacional de Identificación y Estado Civil*) and the Ministry of Health (MINSA, *Ministerio de Salud*). Since 2017, death records are also publicly available on the National Death Registry Information System (SINADEF, *Sistema Informático Nacional de Defunciones*).

The development of SINADEF was an initiative of Peru’s statistics office (INEI), MINSA, and RENIEC with the purpose of enhancing the CRVS system’s quality and coverage (Vargas-Herrera et al., 2018). Furthermore, it represented an opportunity for Peru to revise all the processes involved in the CRVS system, identifying challenges and bottlenecks (Vargas-Herrera et al., 2018).

SINADEF has already brought some positive outcomes to Peru’s CRVS system, even though its implementation is still in progress. First, the system provided a unique database for the whole process of death registration, replacing multiple databases that required additional effort from MINSA during the compilation stage (Vargas-Herrera et al., 2018). Second, the initiative provided a new online cause-of-death classification system and promoted training programs for health workers to improve cause-of-death classification in the country (Vargas-Herrera et al., 2018).

There is strong evidence that these approaches contributed to the reduction of errors in Peru’s cause-of-death information (Miki et al., 2018). Some important challenges remain for the proper functioning of SINADEF and for the accounting of deaths in the health system, such as the unequal distribution of health facilities and human resources throughout the country and the lack of internet infrastructure in remote areas, which result in deaths without the supervision of doctors or without certification (Organisation for Economic Co-operation and Development, 2017; Vargas-Herrera et al., 2018).

For this work, age and sex mortality profiles could be retrieved from the microdata of MINSA for the period 1994–2018 and from RENIEC for the period 2016–2019. Both databases were provided by INEI upon request by e-mail. Furthermore, records of total deaths from 2005 to 2019 by sex from RENIEC are publicly available.^3^

#### ENDES surveys

Since 1986, INEI has been responsible for the ENDES (*Encuesta Demográfica y de Salud Familiar*) survey, a household survey under the model of the Demographic and Health Surveys (DHS) Program (INEI, 1986–2019). ENDES was conducted every 4 or 5 years (1986, 1991, 1996, 2000, 2004); from 2004 on, it was done every year. Through all these years, the ENDES survey has been an important tool for monitoring and evaluating Peru’s population health, which is key to public policy design. More information on the questionnaires and sample size are presented in Appendix A.

For mortality estimation, specifically, ENDES investigates information on birth and sibling histories of women of reproductive age. Also, since 2017, a section on household deaths was included in the survey. This last approach has the advantage of allowing for computing mortality rates by age and sex using death counts and exposed population from the same source for 5 years before the survey.

The microdata of the ENDES surveys (1996, 2000, 2004–2019) are publicly available on INEI’s website.^4^

### Summary of available mortality data sources in Peru

In Table 1, we present a summary of the main sources of death information in Peru and provide some details on the type of geographic disaggregation and age groups available. We have compiled and analyzed all the possible data sources available in the two intercensal periods to evaluate the consistency across databases. We computed death counts (numerator of mortality rates) from MINSA microdata for the years 1994–2018 (MINSA)^5^ by region, age, and sex. Exposed population counts (denominator of mortality rates) by region, age, and sex were collected from national censuses of 1993, 2007, and 2017 (INEI, 1993, 2007, 2017). These two data collections are used to estimate DRRC using death distribution methods (DDM) implemented in the R package **DDM** (Riffe et al., 2017). Afterwards, we computed adjusted and unadjusted life table functions for adult population, namely, death probability at the age range 15 to 49 ($_{35}q_{15}$) and life expectancy at 15 ($e_{15}$). Finally, we compared these measures with official estimates from the United Nations (2019c), the Global Burden of Disease Study from the Institute for Health Metrics and Evaluation (2019), and estimates from ENDES surveys.

**Table 1:** Official data sources for mortality information and disaggregation characteristics, Peru

Name	Time series	Age	Source	Note
MINSA	1986–2017	Single age	–	MINSA publications available since 1961 (microdata from 1986 to 2016)
RENIEC	2005–2019	5-year age groups 2016–2017 only for deaths registered digitally	–	Only available by age after 2016
SINADEF	2017–2019	Single age	MINSA, RENIEC	First data information available in 2017
ENDES	1986–2019	Single age	INEI	DHS type survey, annual period from 2004

### Reference estimates

#### World Population Prospects

World Population Prospects (WPP) consists of population estimates and projections and demographic indicators prepared by the United Nations Population Division (UNPD) for each country and for groups of countries. The estimates of Peru and other Latin American countries are prepared in collaboration with the Latin American and Caribbean Demographic Centre (CELADE) at the Economic Commission for Latin America and the Caribbean. The most recent estimates were published in the 2019 revision and are publicly available online;^6^ it consists of demographic estimates from the 1950s to 2020, and projections are available from 2020 to 2100 (United Nations, 2019b). WPP 2019 estimates (mortality rates, population counts, age-specific fertility rates, and net migration) can also be retrieved through the R package **wpp2019** (United Nations, 2020).

For Peru, the 2019 revision used multiple data sources to derive mortality rates and life expectancy trends, such as sibling deaths from Demographic and Health Surveys (DHS), deaths by age and sex from Ministry of Health records through 2015 adjusted for underregistration using the growth balance method, and official estimates from INEI through 2007, among others (United Nations, 2019a).

For the current work, WPP 2019 life table estimates of Peru are used for the following periods: 1990–1995 (mid-period: 1993), 1995–2000 (1998), 2000–2005 (2003), 2005–2010 (2008), 2010–2015 (2013), and 2015–2020 (2018).

#### Global Burden of Disease

The Global Burden of Disease (GBD) study, coordinated by IHME, investigates demographic and health indicators for all countries in the world (Wong, 2020). The most recent study, published in 2019, compiled life tables for 204 countries, territories, and subnational areas. The data, which contain estimates of life expectancy ($e_x$) and probability of death ($q_x$) by age and sex, are publicly available on the Global Health Data Exchange website.^7^ The GBD project uses the data from the Ministry of Health, censuses, and household surveys as inputs from Peru.^8^ In general, life table functions are estimated by combining adult mortality estimation—adjusted by death registration completeness levels through death distribution methods (DDM)—and under-5 mortality rates (Wong, 2020). In this paper, GBD life tables estimates for Peru from 1993 to 2019 are used for comparison with other estimates.

#### Latin American Mortality Database

The Latin American Mortality Database (LAMBdA) contains information on population and mortality for 19 Latin American countries. LAMBdA also provides life table estimates adjusted for completeness of death registration, age misstatement, and ill-defined causes of death (Palloni et al., 2014). The inputs for LAMBdA estimates are obtained from national census offices, the World Health Organization Mortality Database, and United Nations Demographic Yearbook (Palloni et al., 2014).

We use life table estimates available in the LAMBdA database for Peru from 1990 on (2000 and 2008 life tables for males and females) for a comparison of adult mortality estimates.

## Methods for adult mortality assessment

In this paper, different methods are used to assess adult mortality in Peru. The methods applied vary by data source, given the methodological possibilities of each data set. The methods range from the death distribution methods using two censuses to assess the relative completeness of death registries in relation to census data to sibling histories and the question of recent deaths in the household using the Peruvian Demographic and Health Surveys (INEI, 1986–2019). In this section, all the methods are explained.

### Death distribution methods

Death distribution methods (DDMs) take death and population age and sex distributions and link them through growth rates and mathematical identities to evaluate the relative completeness of death registration or death counts in relation to censuses (Hill, 1999). Three of these methods are largely used in DRRC estimation: (1) generalized growth balance (Brass, 1975; Hill, 1987); (2) synthetic extinct generations (SEG) (Bennett and Horiuchi, 1981); and (3) the adjusted version of the synthetic extinct generation method corrected by relative census coverage estimated from the general growth balance method (combined GGB–SEG) (Dorrington et al., 2008; Hill et al., 2009). The rationale of each of these methods is briefly described in the following paragraphs.

#### Generalized growth balance method

Brass (1975) proposed the growth balance equation to assess population completeness in stable populations closed to migration. This equation states that the entry rate at each age group *x* and over ($N(x)/N(x+)$, where *N*(*x*) is number of members to age group *x* and $N(x+)$ and members of age groups *x* and over is equal to the constant growth rate *r* (stability assumption) plus the exit rate from the population aged *x* and over ($D(x+)/N(x+)$, where $D(x+)$ is the number of deaths to population aged *x* and over. Considering a death record completion level *C* and observed deaths $D^*$, we can estimate DRRC level by the ratio $D^*(x+)/D(x+)$.

This method, which was further extended by Hill (1987), is known as the generalized growth balance (GGB) method, to overcome the assumption of stable population, since most of the countries in which DDMs are used are still undergoing demographic transition processes with varying rates of population growth over the years. The extended method uses two-census populations by age and sex and death counts over the intercensal period and links these two measures through intercensal age-specific growth rates ($r(x+)$) and relative intercensal population coverage $k_1/k_2$, as stated in Eq. 1. The completeness *C* is estimated by fitting a linear regression curve between $N(x+) - r(x+)$ and $\frac{D^*}{N(x+)}$ points and computing the slope of the fitted curve.

1 $$\begin{aligned} \frac{N(x)}{N(x+)} - r(x+) = \frac{1}{C}*\frac{D^*}{N(x+)} + \frac{k_1}{k_2} \end{aligned}$$

Although not assuming a stable population, the method still assumes that population is closed to migration (it can be further adapted to allow for migration; see Hill and Queiroz (2010)), relative census coverage is the same for all ages, and the completeness of reporting of deaths is the same for all ages, above a minimum age (in this paper, age 15). These assumptions will be discussed further in the section dedicated to the combined GGB–SEG method.

#### Synthetic extinct generation method

The SEG method uses age and sex deaths distribution (*D*(*x*)) over a two-census period and the age-specific growth rates (*r*(*x*)) between those censuses to construct an estimated stationary population with distribution ${\hat{N}}(x)$ by summing up the deaths recorded in the period weighted by the age-specific growth rates (Bennett and Horiuchi, 1981). The mathematical identity to estimate ${\hat{N}}(x)$ is presented in Eq. 2.

2 $$\begin{aligned} {\hat{N}}(x) = \int _x^w{ D^*(y) \exp {\Big [ \int _x^y{ r(z) }{\text{d}}z \Big ] } }{\text{d}}y \end{aligned}$$

Hence, the completeness of death counts can be estimated by comparing the constructed synthetic population counts with the observed counts from population censuses:

3 $$\begin{aligned} C = \frac{{\hat{N}}(x)}{N(x)}. \end{aligned}$$

Beyond assuming that population is closed to migration, the SEG method assumes that death completeness is age-independent after a minimum age, there is no substantial age and sex misreporting, and there is no difference between censuses enumeration (Bennett and Horiuchi, 1981, 1984).

#### Combined GGB–SEG method

The adjusted version of the SEG method—also known as the SEG–delta method (Dorrington et al., 2008) or combined GGB–SEG method (Hill et al., 2009)—uses the intercensal relative enumeration coverage denoted by $k_1/k_2$ of the GGB method to adjust either population from the first or second census and overcome biases introduced by the assumption of absence of census omission (Hill, 1987; Hill et al., 2009). The GGB–SEG and SEG and the method are the best fit for the Peruvian census and the results are presented here.

The estimates of death registration completeness were computed using the R package **DDM**, developed to calculate the relative completeness of CRVS using DD methods with population counts in two points in time (Riffe et al., 2017).

These methods have been widely used in demography, and the violation of assumptions has been tested in different simulation exercises (e.g., Dorrington et al., 2008; Hill et al., 2009; Murray et al., 2010; Palloni et al., 2016). All three methods have been shown to be sensitive when migration, age misreporting and constant age completeness, and underenumeration levels assumptions are violated (Dorrington et al., 2008; Hill et al., 2009). In general, the GGB method is more sensitive for age misreporting and differential death coverage by age, and the SEG method is highly affected by migration (Dorrington et al., 2008; Hill et al., 2009). The SEG method is particularly sensitive to significant changes in census underenumeration; the adjusted version of the method, which adjusts relative coverage of censuses computed by GGB, represents a feasible solution to that issue (Dorrington et al., 2008).

The selection of the age range to compute DRRC has been used to overcome possible violations of the methods’ assumptions (Hill et al., 2009; Hill, 2017), especially those related to the migration assumption. Net migration estimates can be used for adjusting population counts in DDM, but this approach should be considered with caution, since net migration estimates are difficult to precise (Moultrie et al., 2013). However, it is still recommended to take into account the possible biases generated when these assumptions are not met. The migration issue is generally treated by adjusting the selection criteria of age groups to compute DRRC levels. Hill et al. (2009) recommends computing the average of GGB and SEG methods using the age range 30+ and 65+ when migration is seen to affect the estimation procedure—it is not recommended to use much older age groups (75+) due to age-misreporting issues. Other approaches consider the use of a hybrid version of GGB and SEG methods: Murray et al. (2010) suggest using SEG for age range 55 to 80, GGB for 50 to 70, and GGB–SEG for 50 to 70, Hill (2017) recommends the age range from 5 to 65 for GGB and 50 to 70 for SEG; and Glei et al. (2019) use age groups 5 to 65, 30 to 65 and the optimum age selection range retrieved by the R package **DDM**, based on the minimization of the root mean square error of estimates.

In this paper, different age ranges are used, and the sensitivity of the methods’ results regarding the age ranges used are evaluated. More specifically, we computed multiple DRRC estimates from a total of 20 age group combinations from 15 to 19 up to ages 75 to 79, considering at least eight adjacent age groups. The minimum and maximum values of these 20 estimates are presented as sensitivity analysis intervals. Although specific age groups could be used considering the ages less likely to be influenced by migration in order to have more exact results of the method, in this paper we present both the minimum, and maximum correction possible using the combined GGB–SEG and SEG methods to shed light on the possible sizes of the adult mortality gap in life expectancy at age 15.

In Latin America, these methods have been frequently used to assess the levels of relative completeness of CRVS data (Palloni et al., 2016) and have shown reliability in mortality assessments of developing countries (Hill, 2017). Recent country-specific papers have been published to evaluate the relative completeness of CRVS data from Guatemala (Hill, 2003), Peru (Piscoya-Diaz and Queiroz, 2010), Brazil (Queiroz et al., 2017, 2020a, 2020b), Costa Rica (Glei et al., 2019), and Ecuador (Peralta et al., 2019).

### Direct estimates of adult mortality using survey data

#### Recent deaths in the household

In the Peruvian ENDES surveys of 2018 and 2019, the respondents were asked about age at death, sex, and date of death of household members who had died in the years before the survey.^9^ These mortality questionnaires, usually applied in national censuses, provide information for computing mortality profiles, which are helpful for understanding age and sex differentials even when the resulting mortality level is not accurate. Furthermore, ENDES has the advantage of providing both the numerator (deaths) and denominator (exposed population) of age-specific mortality rates from the same data source (Moultrie et al., 2013).

One limitation of mortality data collected from surveys is that it is largely sensitive to the sample size, since deaths are rare events (Moultrie et al., 2013). We considered the deaths that occurred within the 5-year interval prior to the survey reference date to minimize problems with sample size and to obtain smoother estimates. Therefore, the periods 2013 to 2017 (mid-period: 2015.5) and 2014 to 2018 (mid-period: 2016.5) were used as reference periods for estimates from the ENDES surveys of 2018 and 2019, respectively.

#### Adult mortality estimation from sibling histories

In countries with defective or incomplete death registration systems, estimates of mortality are usually provided through indirect methods by population censuses or by specific questionnaires in household surveys. The collection of sibling histories has been widely used by the DHS program to provide estimates of adult and maternal mortality in these countries (Stanton et al., 1997; Masquelier, 2013).

From sibling histories data, we can also retrieve direct estimates of adult mortality. The computation of population exposure demands detailed information from siblings, including sex, age, birth date, date of death, and age at death (if deceased) (Stanton et al., 1997; Moultrie et al., 2013). With this set of information, age-specific death rates for previous years are estimated through dividing the death counts over a specific period by the population exposure. The population exposed to the risk of dying is computed by summing up person-years’ contributions of siblings who died and survived over the period (Moultrie et al., 2013).

In the Peruvian ENDES survey, sibling histories were collected in module 73 of the questionnaire, which is specific to maternal mortality assessment. Women aged 12 to 49 were asked about contraceptive use, reproductive history, and survival history of brothers and sisters. Since ages of siblings are usually close to the age of the respondent, estimates of adult mortality probabilities for ages higher than 50 are likely to be biased (Moultrie et al., 2013). Therefore, we focus our mortality probability estimates on ages 15 to 50 ($_{35}q_{15}$).

Despite the straightforward approach, the death probabilities constructed from sibling histories have some important issues that may result in biased estimates. For instance, we may cite the underreporting of deaths (especially for older respondents), which underestimates death probability results (Moultrie et al., 2013), the presence of sibling duplicates, and the small sample sizes of surveys (Masquelier, 2013). Recent findings have also provided evidence of errors of misreporting siblings’ ages and dates (Helleringer et al., 2014a, 2014b; Masquelier et al., 2021).

In this analysis, we tried to minimize the errors brought by omitted deaths by computing estimates of adult mortality for periods up to 20 years before the survey’s reference date. Furthermore, we used multiple sets of sibling histories (from surveys from 2009 to 2019) to evaluate the consistency across estimates (Moultrie et al., 2013). Nevertheless, we must highlight some caveats and warnings regarding the sibling histories collection of the ENDES survey in Peru. The variables of deceased siblings’ date of birth and date of death, used to retrieve population exposures, were all missing in the surveys’ microdata of 2009, 2012, 2013, 2014, 2015, 2016, 2017, 2018, and 2019. Thus, the dates were estimated from the variables on age at death, years since siblings’ death, and date of interview. Also, data for the years 2016 and 2018 presented high missing counts for the sampling weights of female respondents (96% and 94% missing, respectively), and the information of sibling histories for these 2 years were not considered in the analysis. For the other years ranging from 2009 to 2019, the sampling weights of female respondents did not present missing values (detailed information on ENDES’s sample size and missing values for the specific variables are available in Appendix A).

The adult mortality estimates from sibling histories were done using the R package **demogsurv**, developed to calculate demographic measures using household survey data (Eaton and Masquelier, 2021).

## Results

### Sex differentials in death registration completeness

#### Health records vs civil registration

The differences in the total number of deaths available from the two main data sources (MINSA and RENIEC) can be observed in Table 2. Between 2005 and 2011, the difference between deaths registered in the civil registry and in the Ministry of Health ranged from 8.9 to 27.2%. This relative difference increased up to 2016, when the civil registry recorded 49.6% more deaths than the Ministry of Health. The differences by sex in this period are very similar, with deaths of males registered in the civil registry system usually being slightly more than deaths of females (with the exception of the years 2012 and 2015). On average, from 2005 to 2017, MINSA had 24.2% and 27.1% more registered deaths than the MINSA database. From 2010 on, the civil registry data present significant increases in the number of deaths registered from 1 year to the next, which can be a result of efforts to improve the data recollection. The year 2017 presents an unusual result for the MINSA data, with the difference between the two sources decreasing almost 20%. The increase in the MINSA registers in 2017 was slightly higher for females than for males; the difference for males went from 49.9% in 2016 to 25.1% in 2017 and for females from 49.5 to 22.4%, with the difference between the registers in the two sources decreasing by 27%.

The fluctuation observed in 2016–2017 might have been influenced by the implementation of the SINADEF system in its initial stages. The change in procedure and the establishment of online protocols for death registration may have affected the data completeness, since some areas of the country lack the infrastructure to implement these online systems (Vargas-Herrera et al., 2018).

In general, we expect lower numbers of deaths in health records (MINSA) than in civil registries (RENIEC) if higher death counts occur outside of the health systems. Notwithstanding this, if the registration of deaths is very low in the country, or the civil registration office does not offer, for example, an information system with a nationwide database, health records can be higher than the civil registry records. In Latin America, few countries have information technology systems in which the standardization and harmonization of birth and death registries are done at the moment of the event registration, resulting in a single and harmonized database, as in Chile, for example. Brazil and Mexico, such as Peru, also have two databases for death registration: one from civil registration records and the other from health records. Whether the health records are higher or lower varies across countries and considerably within countries, depending on different factors. Brazilian Institute of Geography and Statistics recently developed a matching procedure for the two databases to investigate the differences between them at the municipality level (IBGE, 2018).

**Table 2:** Difference between the number of deaths published by the Ministry of Health and the civil registry by year and sex, from 2005 to 2018, Peru

Year	Females			Males			Total		
	MINSA	RENIEC	% Diff	MINSA	RENIEC	% Diff	MINSA	RENIEC	% Diff
2005	41,052	47,739	16.3	47,652	55,468	16.4	88,704	103,207	16.3
2006	38,117	48,709	27.8	44,503	56,365	26.7	82,620	105,074	27.2
2007	40,411	48,937	21.1	47,084	58,312	23.8	87,496	107,249	22.6
2008	42,279	49,184	16.3	49,011	58,916	20.2	91,290	108,100	18.4
2009	44,238	50,847	14.9	51,483	59,964	16.5	95,722	110,811	15.8
2010	45,936	49,963	8.8	53,398	58,215	9.0	99,334	108,178	8.9
2011	44,669	54,516	22.0	52,183	63,940	22.5	96,852	118,456	22.3
2012	45,266	55,536	22.7	52,720	64,116	21.6	97,989	119,652	22.1
2013	45,794	58,173	27.0	52,822	67,408	27.6	98,616	125,581	27.3
2014	44,673	61,363	37.4	51,787	71,467	38.0	96,460	132,830	37.7
2015	44,643	62,850	40.8	51,597	72,008	39.6	96,240	134,858	40.1
2016	45,310	67,721	49.5	51,918	77,800	49.9	97,241	145,521	49.6
2017	57,192	70,011	22.4	63,950	80,021	25.1	121,142	150,032	23.8
2018	58,952	70,766	20.0	66,638	80,924	21.4	125,590	151,690	20.8
^a^2019	53,107	72,354	36.2	61,332	82,067	33.8	114,449	154,421	34.9

Percent difference computed by 100 * (RENIEC/MINSA − 1)

^a^MINSA 2019 deaths are not available to date. So, 2019 MINSA values correspond to 2019 SINADEF’s online death record values

The RENIEC data present more information and would be ideal to analyze the evolution of the sex gap in Peru over the last decades. However, the data covering the period of three censuses, disaggregated by age and sex, are available only for MINSA—as mentioned in the data section. The RENIEC data are available by age and sex only for 2016 on. In this regard, we use the DDM method to estimate the relative completeness of the MINSA data by sex and the 2016, 2017, 2019, and 2019 RENIEC data to validate the estimated sex gap in adult mortality using the MINSA data and DDM method.

#### Death registration completeness by sex and region

Tables 3 and 4 present the results of completeness of death records from the Ministry of Health using the combined GGB–SEG and the SEG methods for males and females for the 1993–2007 and 2007–2017 census periods. We computed the estimated sensitivity analysis intervals with the minimum and maximum values computed from the 20 possible age selection combinations we presented in the methodological section and their respective mean values.

**Table 3:** Estimated death registration completeness for adult deaths with the two-census SEG and combined GGB–SEG methods in Peru and its regions—1993–2007

Region	Males		Females	
	SEG	GGB–SEG	SEG	GGB–SEG
Amazonas	0.32 (0.28–0.35)	0.50 (0.47–0.53)	0.33 (0.27–0.37)	0.62 (0.57–0.66)
Ancash	0.39 (0.37–0.40)	0.48 (0.44–0.51)	0.35 (0.31–0.38)	0.53 (0.50–0.56)
Apurimac	0.52 (0.47–0.54)	0.65 (0.59–0.76)	0.48 (0.42–0.52)	0.76 (0.70–0.81)
Arequipa	0.81 (0.79–0.83)	0.94 (0.84–1.06)	0.85 (0.83–0.86)	0.93 (0.87–0.99)
Ayacucho	0.37 (0.36–0.38)	0.32 (0.29–0.38)	0.35 (0.33–0.36)	0.40 (0.37–0.42)
Cajamarca	0.40 (0.36–0.42)	0.56 (0.50–0.63)	0.40 (0.35–0.44)	0.64 (0.60–0.68)
Callao	0.89 (0.88–0.90)	0.97 (0.91–1.00)	0.91 (0.90–0.93)	0.91 (0.83–0.95)
Cusco	0.67 (0.62–0.70)	0.85 (0.78–0.97)	0.68 (0.62–0.73)	0.97 (0.92–1.00)
Huancavelica	0.36 (0.35–0.37)	0.38 (0.35–0.43)	0.36 (0.33–0.39)	0.51 (0.47–0.54)
Huanuco	0.62 (0.57–0.65)	0.81 (0.74–0.94)	0.61 (0.54–0.68)	0.99 (0.90–1.07)
Ica	0.86 (0.84–0.88)	0.96 (0.94–1.02)	0.87 (0.84–0.89)	1.01 (0.97–1.10)
Junin	0.65 (0.61–0.67)	0.79 (0.73–0.89)	0.60 (0.55–0.65)	0.86 (0.83–0.88)
La Libertad	0.76 (0.74–0.78)	0.83 (0.78–0.94)	0.76 (0.72–0.78)	0.90 (0.85–0.99)
Lambayeque	0.82 (0.77–0.85)	0.98 (0.91–1.13)	0.82 (0.76–0.86)	1.06 (0.99–1.19)
Lima	0.83 (0.81–0.84)	0.92 (0.86–0.95)	0.86 (0.85–0.87)	0.93 (0.91–1.00)
Loreto	0.33 (0.31–0.35)	0.44 (0.41–0.47)	0.33 (0.30–0.36)	0.51 (0.48–0.53)
Madre de Dios	0.68 (0.65–0.75)	0.57 (0.39–0.68)	0.91 (0.88–0.99)	0.77 (0.54–0.91)
Moquegua	0.73 (0.72–0.74)	0.79 (0.69–0.89)	0.77 (0.77–0.78)	0.84 (0.75–0.91)
Pasco	0.52 (0.51–0.53)	0.58 (0.47–0.66)	0.51 (0.46–0.56)	0.79 (0.67–0.87)
Piura	0.71 (0.67–0.74)	0.87 (0.81–0.96)	0.68 (0.63–0.72)	0.89 (0.84–0.94)
Puno	0.73 (0.71–0.74)	0.76 (0.67–0.89)	0.68 (0.65–0.71)	0.82 (0.78–0.88)
San Martin	0.52 (0.49–0.54)	0.66 (0.63–0.69)	0.57 (0.55–0.59)	0.69 (0.66–0.71)
Tacna	0.82 (0.78–0.84)	0.99 (0.91–1.05)	0.94 (0.93–0.95)	1.04 (0.92–1.11)
Tumbes	0.80 (0.77–0.82)	0.92 (0.85–1.02)	0.85 (0.83–0.87)	0.99 (0.94–1.02)
Ucayali	0.65 (0.62–0.67)	0.78 (0.72–0.82)	0.68 (0.66–0.71)	0.82 (0.77–0.87)
Peru	0.68 (0.65–0.70)	0.80 (0.76–0.85)	0.68 (0.65–0.71)	0.84 (0.81–0.87)

Format: [mean value (sensitivity analysis interval)]. Sources: Estimated from Ministry of Health data and censuses

**Table 4:** Estimated death registration completeness for adult deaths with the two-census SEG and combined GGB–SEG methods in Peru and its regions—2007–2017

Region	Males		Females	
	SEG	GGB–SEG	SEG	GGB–SEG
Amazonas	0.33 (0.29–0.36)	0.48 (0.45–0.53)	0.33 (0.29–0.35)	0.48 (0.44–0.53)
Ancash	0.47 (0.43–0.49)	0.61 (0.52–0.75)	0.47 (0.43–0.49)	0.61 (0.54–0.72)
Apurimac	0.51 (0.50–0.52)	0.49 (0.44–0.57)	0.51 (0.48–0.53)	0.60 (0.51–0.70)
Arequipa	0.80 (0.77–0.83)	0.68 (0.67–0.69)	0.83 (0.79–0.88)	0.69 (0.65–0.70)
Ayacucho	0.38 (0.36–0.40)	0.48 (0.40–0.60)	0.40 (0.37–0.42)	0.52 (0.44–0.64)
Cajamarca	0.35 (0.30–0.39)	0.58 (0.51–0.66)	0.37 (0.33–0.41)	0.58 (0.52–0.67)
Callao	0.86 (0.83–0.89)	1.04 (1.00–1.09)	0.88 (0.86–0.90)	1.01 (0.97–1.05)
Cusco	0.47 (0.45–0.49)	0.54 (0.50–0.62)	0.47 (0.44–0.49)	0.58 (0.52–0.66)
Huancavelica	0.29 (0.21–0.36)	0.73 (0.53–0.98)	0.33 (0.25–0.40)	0.82 (0.63–1.03)
Huanuco	0.46 (0.39–0.51)	0.78 (0.65–0.92)	0.53 (0.45–0.59)	0.90 (0.78–1.04)
Ica	0.91 (0.90–0.92)	0.87 (0.87–0.89)	0.92 (0.92–0.93)	0.91 (0.87–0.93)
Junin	0.56 (0.49–0.61)	0.89 (0.77–1.08)	0.62 (0.55–0.67)	0.91 (0.81–1.04)
La Libertad	0.73 (0.68–0.77)	0.93 (0.83–1.12)	0.78 (0.74–0.80)	0.89 (0.83–1.01)
Lambayeque	0.87 (0.80–0.91)	1.09 (0.99–1.32)	0.88 (0.81–0.92)	1.13 (1.04–1.30)
Lima	0.71 (0.69–0.74)	0.85 (0.82–0.89)	0.74 (0.73–0.76)	0.84 (0.82–0.87)
Loreto	0.20 (0.16–0.22)	0.35 (0.31–0.39)	0.20 (0.17–0.22)	0.37 (0.31–0.42)
Madre de Dios	0.73 (0.72–0.75)	0.77 (0.56–0.84)	0.78 (0.74–0.84)	0.65 (0.56–0.69)
Moquegua	0.56 (0.52–0.60)	0.76 (0.73–0.77)	0.69 (0.66–0.72)	0.84 (0.80–0.87)
Pasco	0.29 (0.22–0.34)	0.69 (0.60–0.79)	0.38 (0.29–0.44)	0.88 (0.74–1.05)
Piura	0.72 (0.69–0.74)	0.80 (0.76–0.92)	0.72 (0.69–0.74)	0.83 (0.79–0.93)
Puno	0.46 (0.40–0.50)	0.71 (0.57–0.97)	0.48 (0.42–0.53)	0.78 (0.63–1.02)
San Martin	0.44 (0.41–0.46)	0.56 (0.53–0.60)	0.52 (0.50–0.53)	0.58 (0.55–0.62)
Tacna	0.78 (0.76–0.79)	0.86 (0.80–0.89)	0.82 (0.81–0.83)	0.89 (0.86–0.92)
Tumbes	0.53 (0.49–0.56)	0.71 (0.64–0.81)	0.55 (0.53–0.57)	0.67 (0.63–0.71)
Ucayali	0.55 (0.52–0.57)	0.66 (0.63–0.72)	0.59 (0.56–0.62)	0.74 (0.70–0.82)
Peru	0.61 (0.57–0.64)	0.79 (0.72–0.90)	0.64 (0.60–0.67)	0.79 (0.74–0.88)

Format: [mean value (sensitivity analysis interval)]. Sources: Estimated from Ministry of Health data and censuses

The relative completeness of death registration of MINSA data in Peru has shown a stagnant or worsening performance in the period from 1993 to 2017. Female deaths had a mean relative completeness from the GGB–SEG method of 84% in 1993–2007 period (ranging from 81 to 87%), which reduced to 79% in the 2007–2017 period (ranging from 74 to 88%). For males, those values were 80% (ranging from 76 to 85%) and 79% (ranging from 72 to 90%) respectively. Therefore, mean estimates and sensitivity analysis intervals do not suggest significant sex differences in completeness of death registration in MINSA data. The wider sensitivity analysis intervals for the last intercensal period might be related to greater migration flows in the country. The sex difference at the subnational level need to be interpreted with caution due to differential internal net migration flows by sex, which means that the sex difference in the relative completeness estimated might not be real but an artifact of the biases produced by the method due to sex-differences in the net migration flows across regions.

Areas from higher socioeconomic strata (located in the coastal areas of the country) are among those with the highest DRRC levels (Tacna, Lima, and Callao). These results might reflect the unequal distribution of health services, infrastructure, and wealth throughout the country (Huynen et al., 2005).

Although these estimates are similar to Piscoya-Diaz and Queiroz (2010) and Urdinola and Queiroz (2020), with the latter having a DRRC of 77% and 81% for males and females, respectively, for 1993 to 2005, the estimated DRRC differs significantly from the relative completeness of 53.3% estimated by Palloni et al. (2014) for Peru in 2008 for age 5 and above Palloni et al. (2016). The reasons for these differences with Palloni et al. (2014) are not clear and require further investigation.

### Sex differentials in adult mortality

#### Probability of dying between ages 15 and 49

**Fig. 1:**
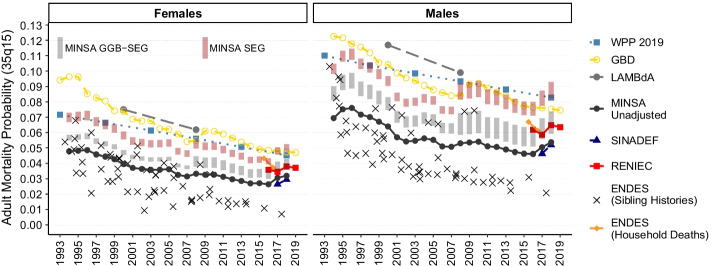
Adult mortality probability—$_{35}q_{15}$—estimates computed from MINSA, SINADEF, RENIEC, ENDES, WPP 2019, and GBD 2019 data by year and sex from 1990 to 2019, Peru. MINSA results were adjusted by GGB–SEG and SEG sensitivity analysis intervals

Adult mortality estimates of $_{35}q_{15}$, which is the probability of dying between ages 15 and 49, are presented in Fig. 1. Overall, WPP 2019, GBD, and LAMBdA estimates present higher levels of adult mortality for both males and females during the entire period analyzed (1990–2019). Estimates from MINSA, SINADEF and RENIEC data are lower than these estimates, as expected, because these data are known to be incomplete, but all series show internal consistency throughout the years. The data bars named “MINSA GGB–SEG” and “MINSA SEG” correspond to the Ministry of Health data series adjusted for relative completeness using the sensitivity analysis interval values presented in Tables 3 and 4, as estimated with the combined GGB–SEG and SEG methods. The series adjusted by SEG method present lower values than the reference estimates, but they are getting closer to GBD and WPP 2019 estimates over the last years than the GGB–SEG method. So, the series adjusted by the SEG method are closer to WPP 2019 and GBD reference estimates. Furthermore, we also present the estimates computed from ENDES survey household deaths and sibling histories. This last method is known for underestimating mortality (Masquelier, 2013) and its results must be seen with caution. Although the levels of these estimates are much lower, they present a trend in time by sex that is consistent with the other estimates.

#### Sex gap in life expectancy at age 15

**Fig. 2:**
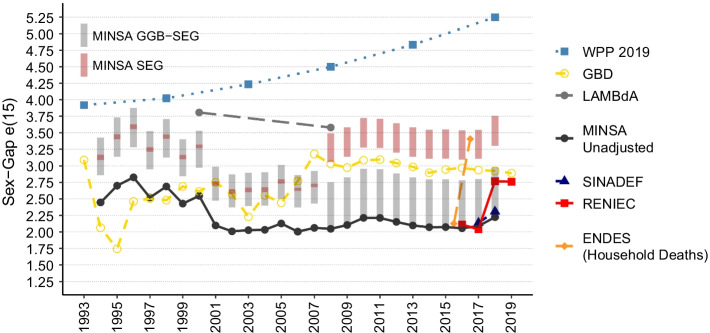
Life expectancy at 15 year sex-gap estimates from MINSA, SINADEF, RENIEC, ENDES, WPP 2019, and GBD 2019 data by year and sex from 1990 to 2019, Peru. MINSA results were adjusted by GGB–SEG and SEG sensitivity analysis intervals

Figure 2 presents the results of estimated sex-gap trends in life expectancy at age 15 in Peru. The sex gap in $e_{15}$ is estimated as the difference between female life expectancy at age 15 and male life expectancy at age 15. WPP 2019 estimates show an increasing trend in life-expectancy sex gap from around 4 years in favor of women in 1990 to 1995 to approximately 5.25 years of female advantage in 2018. These estimates imply strong differential assumptions regarding the completeness of death registration of males and females at ages 15 and above. The other reference estimates do not show a clear trend.

This trend is not followed by our unadjusted estimates using data from MINSA, SINADEF, RENIEC, and the ENDES survey, or the MINSA series adjusted by GGB–SEG and SEG methods. ENDES results of household mortality inquiry present estimates of sex gap ranging from 2.10 in 2015.5 to 3.40 in 2016.5. The estimates using Ministry of Health data (MINSA and SINADEF) show an oscillatory but decreasing trend in the sex gap until the beginning of the availability of SINADEF. The interval estimates obtained by adjusting MINSA data by the sensitivity analysis interval of completeness of death registration levels present results between GBD estimates and unadjusted MINSA estimates from 2010 to 2018, within the range of 2.00 to 3.25 years of sex gap in life expectancy at age 15. Even at the highest levels of estimated DRRC, the estimated sex gap in life expectancy at age 15 is below 3.75 in the last intercensal period.

The age-specific mortality rates, and consequently life expectancy at age 15, estimated using combined GGB–SEG and SEG methods, have some limitations that are important to highlight. First of all, it is not possible to assume that all assumptions of the death distribution methods (population closed to migration, absence of age misreporting, and constant death registration completeness among all age groups) are met. Even though a combination of age ranges was provided to show the possible adjustments to the data and the sensitivity of estimates for breaking these assumptions, for estimating official mortality rates in the country one should define a specific age group selection and implement more testing for the violation of the methods’ assumptions. Also, when estimating mortality rates for age 60 and above, it is important to take into account adjustments for old age mortality misreporting (Preston et al., 1999; Palloni and Pinto-Aguirre, 2011). In this paper, it is assumed that old age misreporting is similar for males and females, and so it would not significantly affect the sex gap in life expectancy at age 15, but it can affect the level of $e_{15}$ for each sex (see Table 8 in Appendix B). The old age adjustment can be driving the differences in the levels of life expectancy encountered in our estimates and LAMBdA and WPP 2019 estimates if the assumptions are not being met. In conclusion, for official life table estimation in Peru, the recommendation is to analyze old age misreporting and to adjust the data accordingly.

## Conclusion

This paper assessed the data available for Peru using different demographic methods to investigate relative death registration completeness of adult deaths by sex and its effects on trends of life expectancy sex differentials at age 15. The complexity underlying mortality estimates and the range of data and methods available can be noted in this work.

Peru has two CRVS data sources: health records from the Ministry of Health (MINSA) for longer age–sex time series; and civil registration records from the civil registration office (RENIEC), which has age data from 2016 on. The two data sources varied considerably in the number of deaths recorded from 2005 to 2019 and users need to have caution when compiling mortality statistics for the country doing a detailed quality assessment of the sources. Recently, MINSA have developed the National Death Information System (SINADEF), a promising database created to unify and harmonize death registration records, enhancing data quality and coverage (Vargas-Herrera et al., 2018).

Besides CRVS data, mortality estimates were also computed using the Peruvian Demographic and Health Survey (ENDES) recent household and sibling histories mortality information. All these results were then compared to publicly available life table estimates collected from the World Population Prospects (WPP 2019 revision), Global Burden of Disease (GBD 2019), and Latin American Mortality Database (LAMBdA). We refer to these three data sources as reference estimates.

Our results and the reference estimates show that CRVS data in Peru are still incomplete over the past decade. Nevertheless, the level assumed for this completeness varied significantly across methods used, which could be noted by comparing the estimates of the probability of dying between ages 15 and 49 with the GGB and GGB–SEG method, also known as the SEG–delta method (Dorrington et al., 2008). However, the completeness estimates are not significantly different between males and females. These results are in tandem with previous studies for the region that did not find striking differences in the levels of completeness by sex (Gonzaga et al., 2018; Peralta et al., 2019).

Although we have not adjusted the mortality data for old age misreporting—so, we assumed old age misreporting was not differentiated by sex—the sex gap in life expectancy estimated with the MINSA data, other unadjusted databases, and the MINSA series adjusted by DRRC estimates does not provide evidence for an increasing sex gap in life expectancy at age 15 in Peru. Our results suggest that the gap in life expectancy at age 15 in Peru in the last decade is between 2 and 3.75 years (considering estimates from both GGB–SEG and SEG), which could be higher because of old age misreporting, contrasting with the 5.25 years presented in the 2019 revision of WPP for the 2015–2020 period.

The paper has also revealed the challenges that emerge when working with CRVS data and mortality estimates and the difficulties in establishing the “one rule fits all” when doing mortality assessments. For instance, the decision regarding which data should be used to estimate mortality rates, RENIEC (civil registry) or MINSA (health records), is not straightforward, because it depends of data availability and data disaggregation. The civil registration system is a multi-stage process that often involves multiple arms of government (Ministry of Health, Ministry of Interior, National Statistics Office) which entails a complex flow of data across the different steps of vital event notification, registration and certification (of cause of death). Having well-structured and interconnected information systems is a challenge and needs consistent institutional integration, data quality assessment and political will for being able to gather all deaths occurring in the country.

The COVID-19 crisis highlighted the great need for robust and timely data collection with subnational breakdowns by age, sex, and cause of death. Moreover, the COVID-19 pandemic reinforced the importance of strengthening CRVS systems for building capacity to monitor the direct and indirect impacts of the disease across the country. Analyzing the quality of CRVS data, either by observing the trends of the registries through time at the national and subnational levels, assessing the completeness of death records with demographic methods, or even using the data for analytical purposes, helps with the diagnosis of problems in death recording at the local level and promotes the enhancement of data capacity and data utility in the country. Discrepancies between health information system and the civil registration data need to be continuously evaluated and studied to guide progressive enhancement to the system. Building up solid and resilient death registration systems is key to advancing the country’s efforts to meet the Sustainable Development Goals.

## Figures and Tables

**Fig. 3:**
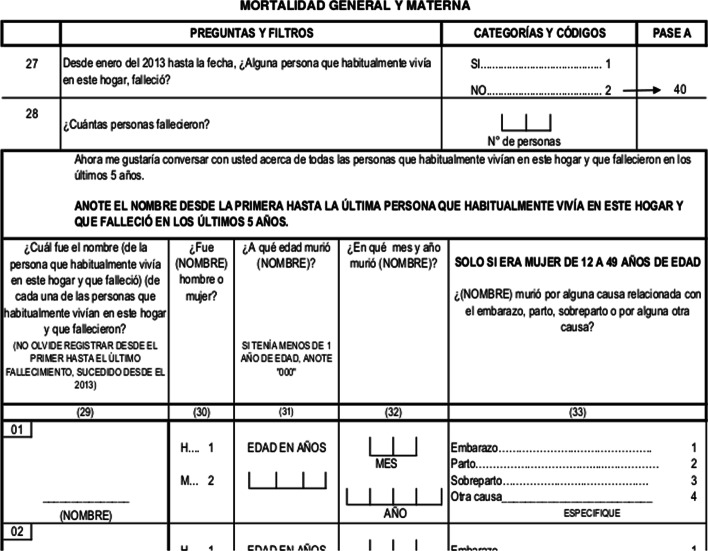
Household mortality questionnaire—ENDES 2018, Peru

**Fig. 4:**
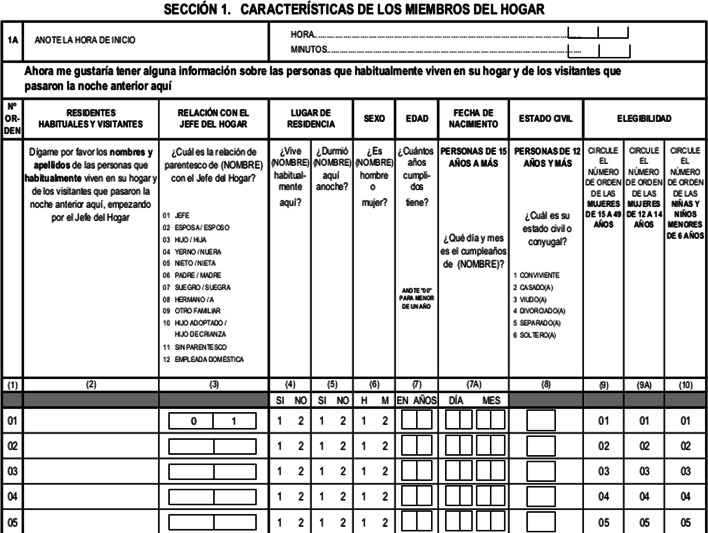
Household members questionnaire—ENDES 2018, Peru

## Data Availability

The data used in this paper are available on INEI’s website (https://www.inei.gob.pe) and from the corresponding author upon reasonable request.

## Appendix A

### ENDES survey—household mortality

The ENDES survey inquired about household deaths that occurred over a specific period in its module 64—household questionnaire. The ENDES survey of 2018 and 2019 had the same sample design and a sample size of 36,760 households. In Figs. 3 and 4, we show parts of the household questionnaire used to retrieve the denominator and numerator counts for computing mortality rates from the survey.

### ENDES survey—sibling histories

The ENDES survey module 73—maternal mortality—questionnaire was answered by females aged 12 to 49. It collected, among other things, birth histories, contraceptive usage, and sibling histories. For adult mortality assessment from the recorded sibling histories, we used the following variables from the ENDES surveys 1996, 2000, 2004–2006, 2009–2019:

- v005: sampling weight for females in the questionnaire for women in reproductive ages (12–49) was used to reconstruct the population profile of this specific subgroup;
- mm1: sex (1—male, 2—female);
- mm2: surviving status of sibling (0—dead, 1—alive, 8—does not know);
- mm3: current age of sibling (values ranging from 0 to 96, 97–97 years and over, 98—does not know);
- mm4: birth date in CMC (century month code) format;
- mm6: years since sibling death (values ranging from 0 to 96, 97–97 years and over, 98—does not know);
- mm7: age of sibling at death (values ranging from 0 to 96, 97–97 years and over, 98—does not know);
- mm8: date of death of sibling in CMC format;
- mm15: sibling’s year of death.

Missing counts for each variable and the number of siblings and sibling deaths reported by female respondents are presented in Tables 5 and 6.

**Table 5:** Number of siblings and sibling deaths recorded in ENDES survey

ENDES year	Number of siblings	Number of sibling deaths	% of siblings in sample after age adjustment
1996	172,417	29,717	99.1
2000	161,003	27,293	99.3
2004–2006 (round 1)	34,850	5955	99.7
2004–2006 (round 2)	69,497	11,519	99.7
2004–2006 (round 3)	107,266	18,189	99.8
2009	128,330	18,746	99.7
2010	123,039	18,429	99.9
2011	118,152	17,536	99.9
2012	123,994	18,512	99.7
2013	117,002	16,103	99.6
2014	123,516	16,967	99.6
2015	178,208	23,274	99.8
2016	162,116	748	3.8
2017	157,603	18,005	99.9
2018	162,903	866	6.0
2019	152,073	16,042	100.0

Peru, 1996, 2000, 2004–2006, 2009–2019

**Table 6:** Percentage of missing values for age and date variables required for mortality assessment from sibling histories in ENDES survey

ENDES year	% of missing or unknown information								
	v005	mm1	mm2	mm3	mm4	mm6	mm7	mm8	mm15
1996	0.0	0.0	0.0	17.6	0.0	66.2	5.3	0.0	0.0
2000	0.0	0.0	0.0	17.1	0.0	54.4	1.9	0.0	54.2
2004–2006 (round 1)	0.0	0.0	0.0	17.1	99.7	14.9	0.2	100.0	85.3
2004–2006 (round 2)	0.0	0.0	0.0	16.6	99.8	11.1	0.5	100.0	89.1
2004–2006 (round 3)	0.0	0.0	0.0	17.0	99.9	8.6	0.3	100.0	91.5
2009	0.0	0.0	0.0	14.7	99.9	11.7	0.6	100.0	89.2
2010	0.0	0.0	0.0	15.0	0.0	11.2	0.1	0.0	89.2
2011	0.0	0.0	0.0	15.0	0.0	10.8	0.3	0.0	90.1
2012	0.0	0.0	0.0	15.0	99.9	12.0	0.4	100.0	88.5
2013	0.0	0.0	0.0	13.8	99.9	14.7	0.4	100.0	86.7
2014	0.0	0.0	0.0	13.8	99.9	19.2	0.7	100.0	82.1
2015	0.0	0.0	0.0	13.1	99.9	18.2	0.3	100.0	82.5
2016	96.2	0.0	0.0	0.5	3.8	17.5	0.3	100.0	82.6
2017	0.0	0.0	0.0	11.4	99.9	18.3	0.0	100.0	81.7
2018	94.0	0.0	0.0	0.5	6.0	24.4	0.0	100.0	75.6
2019	0.0	0.0	0.0	10.5	100.0	20.8	0.0	100.0	79.2

Peru, 2009–2019

## Appendix B

In this appendix, we present the estimates for $_{35}q_{15}$ and $e_{15}$ in selected years in Tables 7 and 8. We chose to report the estimates from the same periods (or close to these periods, when results were not available for them) of the reference estimates of WPP 2019 (1993, 1998, 2003, 2008, 2013, and 2018).

**Table 7:** Probability of dying between ages 15 and 49 estimates ($_{35}q_{15}$) for selected years and data sources

	Year	Males	Females
WPP 2019	1993	0.110	0.072
	1998	0.104	0.066
	2003	0.098	0.061
	2008	0.093	0.056
	2013	0.088	0.051
	2018	0.083	0.045
GBD 2019	1993	0.129	0.094
	1998	0.112	0.080
	2003	0.094	0.068
	2008	0.084	0.055
	2013	0.084	0.054
	2018	0.075	0.047
LAMBdA	2000	0.117	0.075
	2008	0.099	0.062
MINSA^a^–GGB–SEG	1994	0.081–0.090	0.055–0.059
	1998	0.082–0.091	0.051–0.054
	2003	0.064–0.071	0.041–0.044
	2008	0.058–0.073	0.038–0.045
	2013	0.054–0.067	0.032–0.038
	2018	0.059–0.074	0.036–0.043
MINSA^a^–SEG	1994	0.098–0.105	0.067–0.073
	1998	0.099–0.106	0.062–0.068
	2003	0.077–0.083	0.050–0.055
	2008	0.081–0.092	0.050–0.055
	2013	0.075–0.085	0.043–0.047
	2018	0.082–0.093	0.048–0.052
SINADEF	2017	0.046	0.026
	2018	0.052	0.029
RENIEC	2016	0.062	0.036
	2017	0.059	0.035
	2018	0.065	0.038
	2019	0.064	0.037
ENDES (estimated from sibling histories)^b^	1994–1998	0.039–0.096	0.021–0.068
	1999–2003	0.030–0.076	0.009–0.051
	2004–2008	0.030–0.074	0.012–0.040
	2009–2013	0.022–0.074	0.012–0.033
	2014–2018	0.021–0.031	0.007–0.011
ENDES (household deaths)	2015.5	0.067	0.043
	2016.5	0.061	0.037

Peru, 1993–2019

^a^MINSA estimates adjusted by GGB–SEG and SEG methods correspond to the sensitivity analysis interva

^b^ENDES $_{35}q_{15}$ estimates from sibling histories correspond to the maximum and minimum values computed or the respective period

**Table 8:** Life expectancy at age 15 and gap estimates for selected years and data sources

	Year	Males	Females	Gap
WPP 2019	1993	56.23	60.15	3.92
	1998	57.03	61.05	4.02
	2003	57.77	62.00	4.23
	2008	58.56	63.06	4.50
	2013	59.41	64.25	4.84
	2018	60.35	65.59	5.24
GBD 2019	1993	59.47	62.56	3.09
	1994	60.12	62.19	2.07
	1998	61.07	63.55	2.48
	2003	62.90	65.13	2.23
	2008	64.28	67.31	3.03
	2013	63.81	66.79	2.98
	2018	65.12	68.04	2.92
LAMBdA	2000	55.30	59.12	3.81
	2008	56.84	60.42	3.58
MINSA^a^–GGB–SEG	1994	58.88–59.99	62.31–62.85	2.86–3.43
	1998	58.63–59.75	62.34–62.87	3.12–3.71
	2003	60.69–61.68	63.59–64.07	2.39–2.90
	2008	60.69–62.63	63.45–64.64	2.01–2.76
	2013	61.29–63.15	64.12–65.24	2.09–2.83
	2018	60.51–62.47	63.49–64.68	2.21–2.98
MINSA^a^–SEG	1994	57.23–57.98	60.32–61.15	3.09–3.17
	1998	56.97–57.73	60.38–61.20	3.41–3.47
	2003	59.21–59.88	61.81–62.56	2.60–2.68
	2008	58.37–59.60	61.86–62.66	3.06–3.49
	2013	59.05–60.24	62.63–63.38	3.14–3.58
	2018	58.15–59.41	61.91–62.71	3.30–3.76
SINADEF	2017	64.56	66.70	2.14
	2018	63.80	66.11	2.31
RENIEC	2016	60.21	62.32	2.11
	2017	60.33	62.37	2.04
	2018	61.55	64.32	2.77
	2019	61.75	64.51	2.76
ENDES (Household deaths)	2015.5	64.70	66.82	2.12
	2016.5	65.85	69.26	3.41

Peru, 1993–2019

^a^MINSA estimates adjusted by GGB–SEG and SEG methods correspond to the sensitivity analysis interva

## Abbreviations

CELADE: Latin American and Caribbean Demographic Centre
CRVS: Civil registration and vital statistics
DDM: Death distribution methods
DHS: Demographic and Health Survey
DRRC: Death registration relative completeness
ENDES: Encuesta Demográfica y de Salud Familiar (Peru Demographic and Family Health Survey)
GBD: Global Burden of Disease Study
GGB: Generalized growth balance
ICD: International Statistical Classification of Diseases and Related Health Problems
IHME: Institute for Health Metrics and Evaluation
INEI: Instituto Nacional de Estadística e Informática (National Institute of Statistics and Informatics)
LAC: Latin America and the Caribbean
LAMBdA: Latin American Mortality Database
MINSA: Ministerio de Salud (Ministry of Health)
RENIEC: Registro Nacional de Identificación y Estado Civil (National Registry of Identification and Civil Status)
SEG: Synthetic extinct generations
SINADEF: Sistema Informático Nacional de Defunciones (National Death Information System)
UNPD: United Nations Population Division
WPP: World Population Prospects

## Footnotes

1 The word *regions* is used in this work to represent the administrative unit of Peru defined as *Departamentos* in Spanish.

2 Childhood and adulthood are divided here at age 15, as suggested by Hill (2017), since it is close to the inflection age of the mortality curve and mortality up to age 15 can be plausibly assessed using birth histories or summary birth history methods. In this regard, adult deaths are considered deaths that occur from age 15 and up.

3 See http://webapp.inei.gob.pe:8080/sirtod-series/ and https://portales.reniec.gob.pe/web/estadistica/regCiviles.

4 See http://iinei.inei.gob.pe/microdatos.

5 MINSA microdata was not available for the year 1993.

6 See https://population.un.org/wpp

7 See http://ghdx.healthdata.org/gbd-2019.

8 See http://ghdx.healthdata.org/gbd-2019/data-input-sources?locations=123&components=21.

9 In the 2018 survey, the reference starting date was 1 January 2013, and in the 2019 survey it was 1 January 2014. In the 2017 survey, the question was asked, however, the variables are not available in the publicly available database.
